# Editorial: New advances in grit research: A multidisciplinary perspective

**DOI:** 10.3389/fpsyg.2022.967591

**Published:** 2022-08-09

**Authors:** Song Wang, Jiang Jiang, Xin Tang, Fengmei Lu

**Affiliations:** ^1^The Clinical Hospital of Chengdu Brain Science Institute, MOE Key Laboratory for Neuroinformation, University of Electronic Science and Technology of China, Chengdu, China; ^2^Huaxi MR Research Center (HMRRC), Department of Radiology, West China Hospital of Sichuan University, Chengdu, China; ^3^Beijing Key Laboratory of Applied Experimental Psychology, National Demonstration Center for Experimental Psychology Education, Faculty of Psychology, Beijing Normal University, Beijing, China; ^4^Faculty of Educational Sciences, University of Helsinki, Helsinki, Finland

**Keywords:** grit, positive psychology, performance, wellbeing, measurement, individual differences

Grit, a popular research topic in psychology over the past decade, was first introduced in 2007 and defined as a compound personality of perseverance and passion to achieve long-term goals (Duckworth et al., 2007). Many earlier studies have shown the beneficial role of grit in achievement and life outcomes. For instance, higher levels of grit are associated with higher educational attainment, greater school and workplace performance, a healthier lifestyle, better life satisfaction, more positive affect and less negative affect, and lower risks of mental disorders and problematic behaviors (Fernández et al., 2020; Datu, 2021). However, some recent studies have begun to question and re-examine grit, such as the factor structure of grit, its association with other similar constructs, and its predictive ability for outcomes (Tang et al., 2019, 2021; Ponnock et al., 2020; Morell et al., 2021; Jiang et al., 2022). This Research Topic aims to address those controversies and wishes to advance the frontiers of grit research from a multidisciplinary perspective.

He et al. begin this Research Topic with psychometric research on the Short Grit Scale (Grit-S) among 709 Chinese nurses. The authors found that the Grit-S retained the two-factor structure of the original scale, showed adequate internal consistency and test-retest reliability, and had satisfactory convergent validity with the measures of self-control, psychological resilience, and the “big five” personality traits. With regard to the criterion-related validity, the perseverance of effort but not consistency of interest of grit was related to nursing task performance after controlling for age, gender, length of nursing work, and other personality factors. Additionally, the Grit-S exhibited good measurement invariance between nurses in general hospitals and psychiatric hospitals. These results are generally consistent with previous psychometric findings by Grit-S in other Chinese populations (Li et al., 2018; Zhong et al., 2018; Luo et al., 2020), suggesting the usefulness of Grit-S in Chinese nurses.

Four studies in this volume examined the possible psychosocial mechanism of grit to know how it affects individuals' developmental outcomes. A prospective study by Sulla et al. examined the longitudinal role of grit in university students' achievement in online learning settings during the COVID-19 pandemic. They observed that self-efficacy severed as a mediator between grit and course grades, and psychological distress moderated this mediation pathway. Second, Yang and Wu revealed that grit can influence the meaning of life of nurses in three ways: *via* the mediating role of social support, *via* the mediating role of dispositional hope, and *via* the chain mediating role of social support and dispositional hope. Through a questionnaire survey method looking at 2,602 college students, Zhang et al. found grit had a moderating role in the association between perfectionism and depression symptoms. Concretely, grit partly buffered the effects of negative perfectionism on depression and completely buffered the effects of positive perfectionism on depression. Finally, through two studies on college freshmen students, Yang et al. reported that grit had multiple roles in the relation between interpersonal stress and psychological security. Specifically, grit not only mediated the effect of interpersonal stress on psychological security but also played a moderating role in the link of interpersonal stress with psychological security. In summary, these studies suggest the important role of grit in personal development and wellbeing and show new possible psychosocial mechanisms in explaining the effects of grit.

Another four studies delved into the antecedent factors of grit. First, in 1,871 college students from 12 geographically diverse universities in China, Cheung et al. found that grit was negatively predicted by adverse childhood experiences, and the most strongly predictive factors were emotional neglect and abuse and sexual abuse. Second, Imafuku et al. probed the role of maternal grit and parenting style in the development of grit among children aged 3–6 years. Their findings indicated that mothers' grit levels but not parenting styles (i.e., responsiveness and control) were predictive of individual differences in the grit of children. Third, based on 2,839 students across 21 middle schools in Chiang Mai of Thailand, Tangmunkongvorakul et al. investigated the association between social connectedness and grit; they identified a battery of social connectedness factors linked to grit, including parental support, having been told by parents that they had done something bad, having been asked by parents to do homework, interest in school and satisfactory relationship with teachers. Fourth, from a perspective of transpersonal psychology, Agrawal et al. systematically explored the antecedents and consequences of grit in a sample of employed adults. The authors observed that several transpersonal factors (i.e., metacognition, empathy, optimism, and flow) were linked to grit, which in turn enhanced job performance and job satisfaction. Taking those four studies together, they have deepened our understanding of the antecedents of grit, which may be helpful for possible grit intervention designs.

Additionally, an electroencephalography study by Aguerre et al. investigated the neurobiological substrates of grit in 120 young adults with diverse work experiences and educational backgrounds. The study found that participants with higher grit showed lower frontal theta/beta ratios during a learning task involving top-down control processes. Moreover, perseverance of effort was found to be associated with entropy during a task, indicating the task may require more effort and engagement. Importantly, these findings persisted after controlling for demographic variables and impulsiveness, a self-control-related construct that is highly linked with grit (Pan et al., 2021). In line with the previous neural findings on grit (Wang and Li, 2021), those studies suggest that grit may share some unique neurobiological markers.

Finally, Yu et al. focused on a type of domain-specific grit (i.e., math-specific grit) and tested its possible mediating role in the relation between math anxiety and math achievement. In study 1 based on 222 10th-grade students, the authors observed that math-specific grit but not domain-general grit can mediate the linkage of math anxiety with math achievement. This finding was replicated in study 2 in another group of 465 11th-grade students; furthermore, math-specific grit and math-specific procrastination showed serial mediating effects on the relationship between math anxiety and math achievement. Since recent literature has shown that domain-specific grit outperforms domain-general grit in predicting academic achievement (Clark and Malecki, 2019; Schmidt et al., 2019), this research may ignite more studies of domain-specific grit.

As a whole, the papers in this Research Topic take multidisciplinary approaches and provide valuable insights into understanding grit in different research fields. It has to be noted that given the limited data in this volume, it may be premature to draw a conclusion about the major controversies on grit. However, we hope that this volume will stimulate more scientific investigations on grit and hope many of its controversies can be resolved. The findings of this volume also advance the development of psychoradiology, a burgeoning field at the intersection of psychology, psychiatry and radiology (Canario et al., 2021; Lai et al., 2022; Suo et al., 2022; Zhang et al., 2022).

## Author contributions

SW drafted the manuscript, which all authors reviewed and approved for publication. All authors contributed to the article and approved the submitted version.

## Funding

This work was supported by the National Natural Science Foundation of China (Grant Nos. 31800963 and 62006038) and the Sichuan Science and Technology Program (2022YFS0180).

## Conflict of interest

The authors declare that the research was conducted in the absence of any commercial or financial relationships that could be construed as a potential conflict of interest.

## Publisher's note

All claims expressed in this article are solely those of the authors and do not necessarily represent those of their affiliated organizations, or those of the publisher, the editors and the reviewers. Any product that may be evaluated in this article, or claim that may be made by its manufacturer, is not guaranteed or endorsed by the publisher.
